# Active Use and Engagement in an mHealth Initiative Among Young Men With Obesity: Mixed Methods Study

**DOI:** 10.2196/33798

**Published:** 2022-01-25

**Authors:** Alexander Wilhelm Gorny, Wei Chian Douglas Chee, Falk Müller-Riemenschneider

**Affiliations:** 1 Centre of Excellence for Soldier Performance Singapore Armed Forces Singapore Singapore; 2 Headquarters Medical Corps Singapore Armed Forces Singapore Singapore; 3 Saw Swee Hock School of Public Health National University of Singapore Singapore Singapore; 4 Yong Loo Lin School of Medicine National University of Singapore Singapore Singapore; 5 Digital Health Center Berlin Institute of Health Berlin Germany

**Keywords:** mHealth, physical activity, health promoting financial incentives, weight loss maintenance, young men

## Abstract

**Background:**

The effectiveness of mobile health (mHealth) approaches that employ wearable technology to promote physical activity have been the subject of concern due to the declining active use observed in trial settings.

**Objective:**

To better contextualize active use, this study aimed to identify the barriers and enablers to engagement in a tracker-based mHealth initiative among young men who had recently completed a 19-week residential weight loss program.

**Methods:**

A mixed methods study was conducted among 167 young men who had voluntarily enrolled in the national steps challenge (NSC), an mHealth physical activity promotion initiative, following a residential weight loss intervention. A subsample of 29 enrollees with a body mass index of 29.6 (SD 3.1) participated in semistructured interviews and additional follow-up assessments. Quantitative systems data on daily step count rates were used to describe active use. Qualitative data were coded and analyzed to elicit barriers and enablers to microlevel engagement in relation to the NSC, focusing on tracker and smartphone use. We further elicited barriers and enablers to macrolevel engagement by exploring attitudes and behaviors toward the NSC. Using triangulation, we examined how qualitative engagement in the NSC could account for quantitative findings on active use. Using integration of findings, we discussed how the mHealth intervention might have changed physical activity behavior.

**Results:**

Among the 167 original enrollees, active use declined from 72 (47%) in week 1 to 27 (17%) in week 21. Mean daily step counts peaked in week 1 at 10,576 steps per day and were variable throughout the NSC. Barriers to engagement had occurred in the form of technical issues leading to abandonment, device switching, and offline tracking. Passive attitudes toward step counting and disinterest in the rewards had also prevented deeper engagement. Enablers of engagement included self-monitoring and coaching features, while system targets and the implicit prospect of reward had fostered new physical activity behaviors.

**Conclusions:**

Our study showed that as the NSC is implemented in this population, more emphasis should be placed on technical support and personalized activity targets to promote lasting behavior change.

## Introduction

Physical inactivity has been identified as a major risk factor for noncommunicable disease, early mortality, and increasing health care costs [1-3]. An active lifestyle is considered essential to weight loss and weight loss maintenance [4-7]. To improve physical activity, interventions should be tailored, goal-oriented, and multifaceted [8,9]. Mobile health (mHealth) technologies have offered new opportunities to achieve this in clinical care [10], population health [11,12], and consumer wellness settings [13]. Moreover, the mHealth approach is thought to improve access for groups who experience stigma during physical activity, such as persons with obesity [14,15].

Clinical mHealth interventions commonly employ self-monitoring, goal setting coaching prompts, as well as games and competitions to drive the motivation to exercise [16-18]. However, strong evidence is still lacking on the effectiveness of these approaches in young adults [19,20]. Conversely, mHealth programs that feature health promoting financial incentives (HPFIs) have been shown to provide powerful extrinsic motivation in young persons and adults [21-24]. However, the moral underpinnings of offering a reward in return for health behaviors have been the subject of debate [25,26], and several studies have questioned whether HPFIs can produce sustained behavior change [27-30]. A recent paper has, however, characterized the relationship between physical activity behaviors and habits as bidirectional [31]. This means that once initiated by an HPFI [32,33], new behaviors such as activity tracking could reinforce physical activity habits and promote a virtuous cycle that persists even after the incentives have been discontinued.

One such mHealth initiative is the national steps challenge (NSC) which was first launched by Singapore’s Health Promotion Board (HPB) in 2015 [34]. The NSC provided free access to a wrist-worn tracker that measured step counts and heart rate. Health points were awarded for attaining daily activity goals, and these could be converted to shopping or dining vouchers through a smartphone app, the Healthy 365 app. The system also featured back-end data linkages to accommodate 5 popular consumer tracking devices [35]. NSC incentives would lapse after 5 months, but enrollees would retain their trackers and free access to the Healthy 365 app.

In addition to its rollout to the general population, the NSC offered corporate programs to specific population groups. One such example was young men fulfilling national service obligations in the Singapore Armed Forces. During their basic training, all young men with a body mass index of 27.0 kg/m^2^ or greater entered a 5-month residential weight loss program [36,37]. In December 2018, 1 intake of the residential program was offered to enroll in the NSC.

Many mHealth interventions experience a significant decline in active use [38], as reflected in quantitative systems data [39-41]. Decreasing [42] or insufficient engagement [43] have been cited as a possible explanation for such a decline. Current literature conceptualizes engagement with mHealth interventions as experiences and behaviors [44] that may vary between individuals and over time [45]. To better contextualize mHealth engagement, 2 complementary levels have been proposed: an operational microlevel that comprised moment-to-moment use and a strategic macrolevel where behavior change in pursuit of behavioral goals occurred [46].

Specifically in the areas of obesity management and weight maintenance, gaps in our understanding of mHealth use and engagement remain [47]. A review of 23 studies examining technology in the management of obesity highlighted that while user experience was critical to technology acceptance [48], only 2 studies reported user satisfaction as an outcome [49].

The NSC presented a unique opportunity for our team to build on previous studies involving the residential weight loss program [50,51] and to aid our continuous efforts to promote physical activity in this group. In this mixed methods study, we therefore aimed to examine how qualitative engagement in the NSC could account for quantitative active use and discuss how the mHealth intervention might have changed physical activity behavior.

## Methods

### Study Protocol

Our mixed methods study protocol was approved by the Defence Science Organization of Singapore Armed Forces Institutional Review Board, reference 0010/2019. The quantitative component of this study examined longitudinal activity data, while the qualitative component comprised semistructured interviews. Multimedia Appendix 1 summarizes the phases, procedures and products of data collection and analysis [52]. Our methodological orientation was a grounded theory approach that focused on lived experiences of study participants. We employed a concurrent triangulation of methods strategy followed by data integration to coalesce our study findings. Our research team comprised 2 male, normal-weight military physicians, 1 of whom had experience in qualitative methods. Analyses and reporting were guided by a third author with expertise in physical activity promotion.

### Study Population and Recruitment

**Figure 1:**
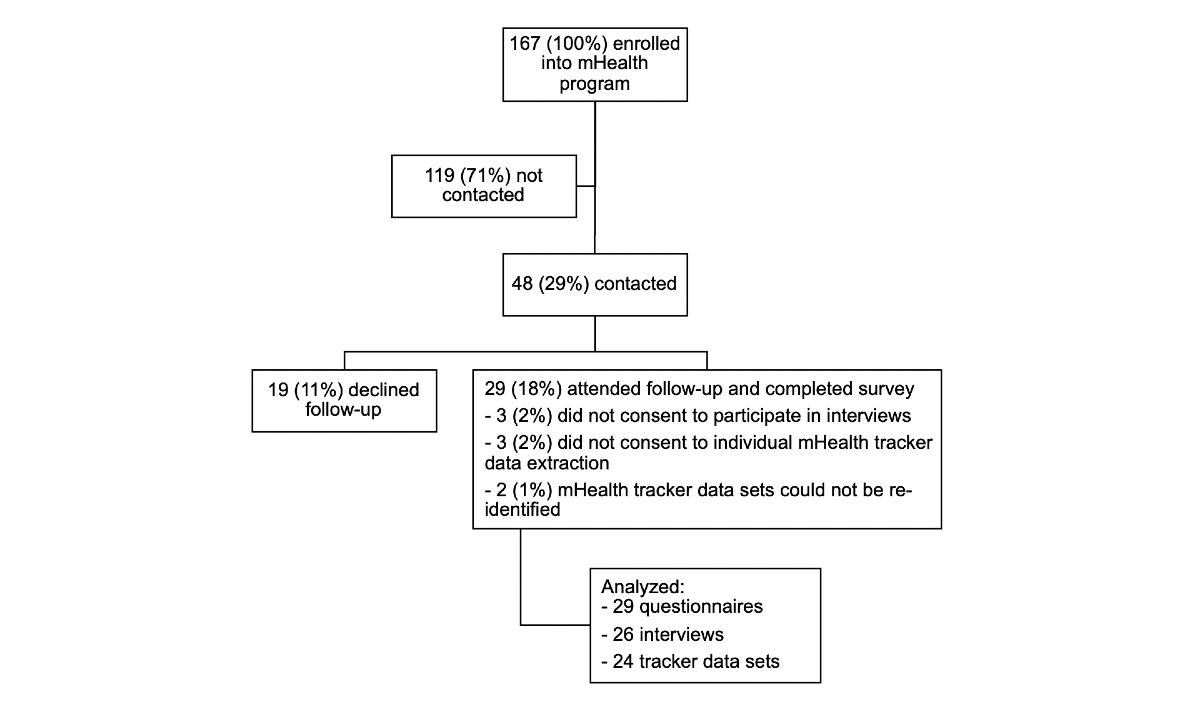
Recruitment for follow-up study, 6 months after enrollment into the national steps challenge.

In November 2018, 3 weeks before the end of the residential weight loss program, all 386 participants were invited to enroll in the NSC Season 4 (2018-2019) on a strictly voluntary basis. A cohort of 167 (43%) young men expressed interest and were issued the NSC tracker 2 weeks later. In June 2019, 6 months after the initial enrollment and 1 month after the official end of the NSC, the research team invited a convenience sample of 48 cohort members working in 6 different large military camp complexes to participate in our study. To be deemed eligible, they had to be at least 21 years old and provide written informed consent to complete a survey, participate in semistructured interviews, and provide their mHealth data (Figure 1). The research team reached out to potential candidates via email to their immediate superiors. Study visits were scheduled for weekday afternoons at the nearest military medical facility. Participation was strictly voluntary, and information would be handled confidentially. No honoraria were paid, but time off from work was granted for the duration of the study visit.

### Quantitative Component

#### Quantitative Data

HPB Singapore provided our research team week-by-week summary data for the 167 cohort members. Active use was defined as a participant who registered at least 1 day with a nonzero step count for a given week. Days with zero steps were treated as missing data and excluded from the computation of average daily step counts. For the subset of consenting study participants, HPB released individually identifiable data that reflected step counts on a day-by-day level. Our questionnaire covered basic demographic information, the Behavioral Regulation in Exercise Questionnaire (BREQ-3) [53-55] and the International Physical Activity Questionnaire Short Form (IPAQ-SF) [56].

#### Quantitative Analysis

We retained HPB’s definition of active use for the 167 cohort members and displayed weekly numbers of active users along with their average daily step counts graphically. To examine individually identifiable active use data more closely, we adopted a threshold of at least 1500 registered steps to indicate a valid day of active use [57]. In this group, nonvalid days of active use were treated as missing data and excluded from further reporting.

### Qualitative Component

#### Interviews

Semistructured interviews followed a topic guide that was developed specifically for this study (Multimedia Appendix 2). Interviewers introduced themselves as medical professionals examining the effects of the NSC, soliciting open feedback, and exploring the lived experience. All interviews were conducted in a private room, recorded digitally, and transcribed by members of the research team.

#### Qualitative Data Management

First, 1 of the authors reviewed handwritten field notes and transcripts and coded key information according to the topic guide. In a second round of coding, interviewees’ experiences using the trackers, synchronizing data, and responding to coaching prompts were coded as microlevel engagement. Interviewees’ attitudes and behaviors that reflected involvement in the behavior change process (eg, related to daily goal setting, accumulation of health points, and redemption of rewards) were coded as macrolevel engagement. In a third round of coding, enablers were identified as circumstances that favored engagement or situations where engagement resulted in positive emotions or perceived benefits. Barriers were identified as circumstances that inhibited engagement or situations where engagement resulted in negative emotions or perceived loss.

#### Data Triangulation, Mixing, and Integration

In a triangulation of methods [58], we produced individual timeline plots to visually validate self-reported active use and categorize the duration of active use. A “short-term user” had accumulated less than 14 weeks of active use while a “sustained user” would have recorded or reported active use that was 14 weeks or longer. BREQ-3 scores, IPAQ-SF outcomes, anthropometric measurements, and barriers and enablers to engagement were reported by category of active use. Anthropometric data were compared using unpaired *t* tests, while ordinal nonparametric BREQ-3 and IPAQ-SF data were analyzed using the Mood median test with *P*=.05 as the chosen level of statistical significance. Integration of findings provided a coherent narrative on active use and engagement before discussing changes in physical activity behavior.

All quantitative analyses were conducted using Stata 13 (Stata Corp LLC). Qualitative data were collated and analyzed using NVivo 12 (QSR International). We used the Consolidated Criteria for Reporting Qualitative Research and the Good Reporting of a Mixed Methods Study checklists when compiling this manuscript [59,60].

## Results

### Quantitative Results

**Figure 2:**
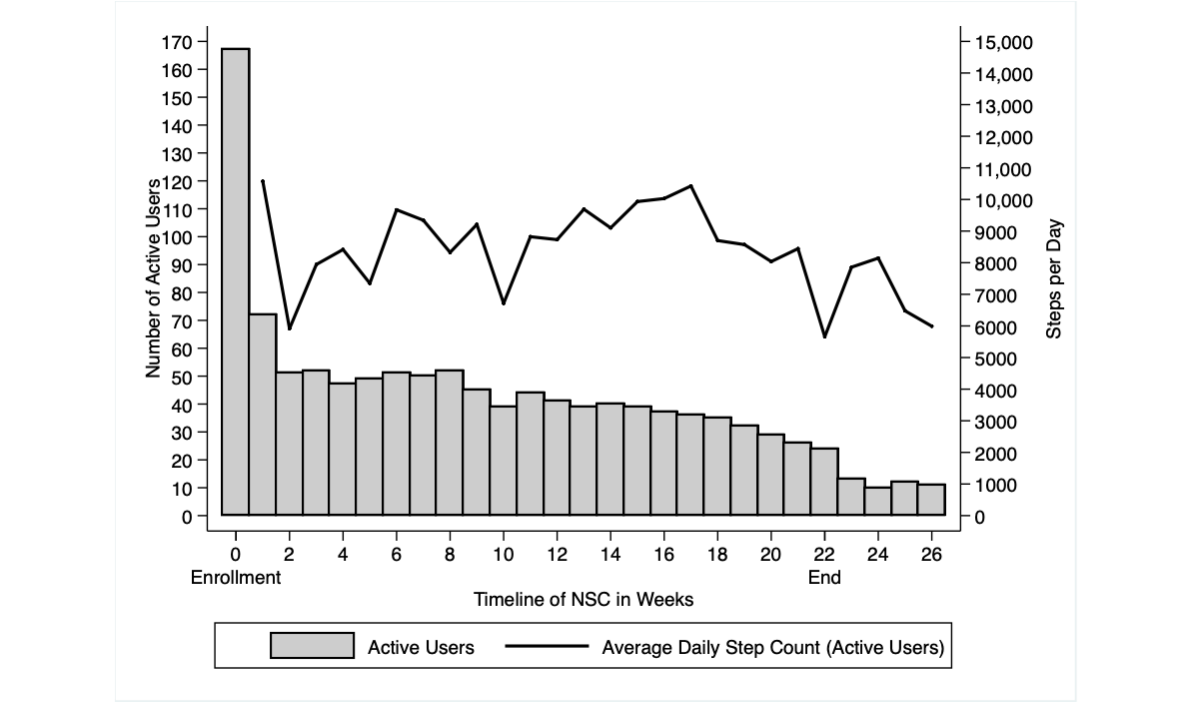
Active users registered in the national steps challenge (gray bars) and their average daily step count (line) by week of enrollment. NSC: national steps challenge.

mHealth system data showed that, in the first week, 72 (43%) of the initial 167 enrolled users were actively recording step counts on a daily basis. On average, these users had walked 10,576 steps per day (Figure 2), excluding days of inactive use when zero steps had been registered. Thereafter, the number of active users declined steadily over the course of the NSC with only 27 (17%) of users recording any steps in the final week.

From the 48 invited users, we recruited 29 participants, 21 to 25 years old, for the follow-up visit at 6 months (Table 1). However, 3 participants (P, U, and X) did not provide informed consent to contribute tracker data, and 2 participants (J and Z) who had provided consent could not be reidentified as registered users on the NSC. A total of 24 tracker data sets were thus available for detailed quantitative reporting and analysis. Of the 1357 person-days with nonzero step counts, 1070 (78.9%) were designated as valid days of mHealth use (Multimedia Appendix 3).

**Table 1:** Descriptive statistics for n=29 study participants.

Characteristics			Values		
			Sustained users (n=13), n (%)		Short-term users (n=16), n (%)
**Ethnicity**					
	Chinese	8 (62)		11 (69)	
	Malay	2 (15)		1 (6)	
	Indian	1 (8)		2 (13)	
	Others	2 (15)		2 (13)	
**Education**					
	Technical or “O” levels equivalent	0 (0)		2 (13)	
	Polytechnic or “A” levels equivalent	13 (100)		14 (88)	
**Smoking**					
	Non-smoker and ex-smoker	9 (69)		14 (88)	
	Smoker	2 (15)		4 (25)	

**Figure 3:**
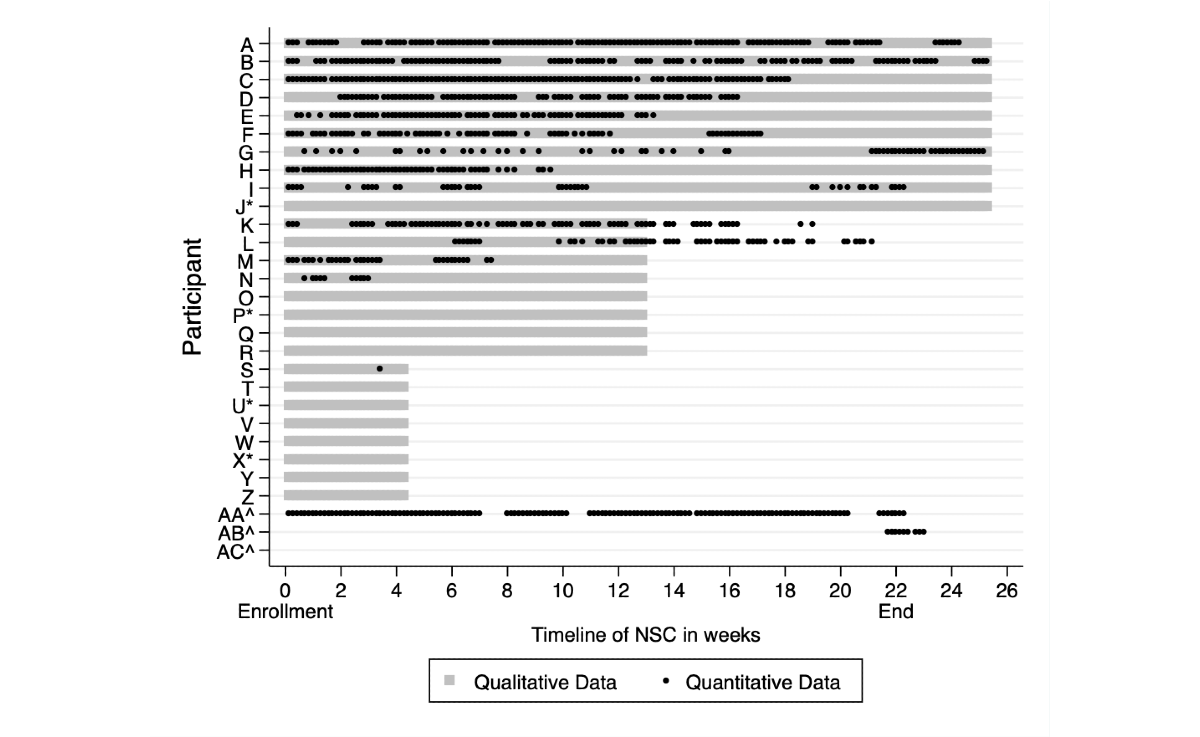
Recorded (black dots) and self-reported (gray bars) duration of active use in n=29 participants; * denotes participants who did not consent to or failed in the retrieval of tracker data; ^ denotes participants who declined to interview. NSC: national steps challenge.

Combined quantitative and qualitative data (Figure 3) allowed us to categorize participants A to L and AA as “sustained users” while participants M to Z, AB, and AC were categorized as “short-term users.” Overall, sustained users expressed a higher level of agreement with BREQ-3 statements in the autonomous spectrum of motivations to exercise (identified, integrated, and intrinsic), greater levels of activity recorded on the IPAQ-SF, and marginally lower levels of weight regain than those interviewees categorized as short-term users (Table 2). These differences, however, were not statistically significant.

**Table 2:** Motivations to exercise, levels of activity, and anthropometric measures for n=29 study participants.

Median measures		Overall (n=29)	Sustained users (n=13)	Short-term users (n=16)	*P* values
**BREQ-3^a^ median score (IQR)**					
	Amotivation	1.38 (1-2)	1.25 (1-1.5)	1.5 (1-2.5)	.26^#^
	External regulation	2.5 (2.25-3)	2.75 (2.5-3)	2.25 (2-3)	.14^#^
	Introjected regulation	3.25 (2.75-3.63)	3.25 (3-3.75)	3 (2.25-3.25)	.14^#^
	Identified regulation	3.75 (3.25-4.13)	3.75 (3.5-4.25)	3.25 (2.75-4)	.28^#^
	Integrated regulation	2.75 (2.25-3.13)	2.75 (2.25-3.5)	2.75 (2.25-3)	.74^#^
	Intrinsic motivation	3.25 (3-3.75)	3.5 (3.25-3.75)	3 (2.75-3.5)	.14^#^
**IPAQ-SF^b^ median time in min/day (IQR)**					
	Sitting	360 (270-540)	360 (300-480)	420 (210-600)	.31^#^
	Walking	130 (50-385)	140 (105-350)	60 (0-420)	.71^#^
	MVPA^c^	15 (0-47)	34 (9-51)	9 (0-30)	.26^#^
**Mean weight measures, kg (SD)**					
	Upon entry into residential program	99.4 (11.0)	97.4 (14.0)	101 (8.0)	.40^
	Upon enrollment into NSC^d^	83.8 (10.4)	81.9 (13.7)	85.4 (6.6)	.38^
	At time of study	89.2 (10.2)	86.8 (12.7)	91.1 (7.7)	.26^
**Mean change in weight, kg (SD)**					
	From entry into residential program to NSC enrollment	-15.6 (4.1)	-15.5 (4.9)	-15.6 (3.4)	.94^
	From NSC enrollment to follow-up study	5.3 (4.5)	4.9 (5.6)	5.7 (3.5)	.61^
**Mean BMI, kg/m^2^ (SD)**					
	Entry into residential program	33.0 (3.2)	32.8 (3.5)	33.2 (2.9)	.72^
	NSC enrollment	27.9 (2.9)	27.6 (3.7)	28.1 (2.3)	.64^
	Follow-up study	29.6 (3.1)	29.3 (3.8)	30.0 (2.5)	.56^

^a^BREQ-3: Behavioral Regulation in Exercise Questionnaire 3.

^b^IPAQ-SF: International Physical Activity Questionnaire Short Form.

^c^MVPA: moderate-to-vigorous physical activity.

^d^NSC: national steps challenge.

^#^Using the Mood median test with the Pearson chi-squared statistic.

^^^Using the unpaired *t* test.

### Qualitative Results

Of the 29 participants, 3 (AA, AB, and AC) declined to participate in the qualitative segment of our study, meaning 26 semistructured interviews comprising a total of 6 hours and 27 minutes of recordings were available for analysis. An overview of barriers and enablers of microlevel and macrolevel engagement is provided in Table 3.

**Table 3:** Overview of barriers and enablers elicited from n=26 interviews.

Barriers and enablers		Sustained users (n=12), n (%)		Short-term users (n=14), n (%)
**Microlevel barriers**				
	Workplace safety regulations requiring clean wrists	1 (8)	3 (21)	
	Removal of hard objects for contact sport	2 (17)	1 (7)	
	Device failure, short battery life, frequent charging	2 (17)	3 (21)	
	Problems performing pairing of tracker with smartphone and problems synchronizing data through cellular network	3 (25)	7 (50)	
	Switch to a new tracker or wearable device	8 (67)	9 (64)	
**Microlevel enablers**				
	Use of tracker as a watch	1 (8)	5 (36)	
	Visualization of cumulative step counts	5 (42)	6 (43)	
	Convenient means of monitoring heart rate and exercise intensity	4 (33)	2 (14)	
	Tracker-based coaching prompts	4 (33)	3 (21)	
**Macrolevel barriers**				
	Passive attitude toward step count tracking	8 (67)	3 (21)	
	Sense of fairness or discomfort tracking incidental physical activity	1 (8)	1 (7)	
	Psychological pressure to make steps count, be active, or attain goals	1 (8)	1 (7)	
	Disinterest in the types of rewards	4 (33)	5 (36)	
**Macrolevel enablers**				
	Redeemed at least one reward	10 (83)	4 (29)	
	Desire to maximize daily health points by adopting NSC^a^ targets	5 (42)	0 (0)	
	Personalized goal setting beyond system targets	5 (42)	0 (0)	

^a^NSC: national steps challenge.

### Barriers to Microlevel Engagement

A multitude of extrinsic and intrinsic factors led to the temporary removal or abandonment of the trackers. Some users overcame technical challenges simply by using the tracker in an offline mode thus forgoing data synchronization and rewards but retaining some of the basic features such as goal setting and self-monitoring. The chief reason for device switching was personal preference, given that more advanced devices generally offered additional functionality. Several users reported difficulty linking their new devices with the Healthy 365 platform, and only 1 sustained user reported that he had accumulated health points using his new tracker.

### Enablers of Microlevel Engagement

Some users had been extrinsically motivated to don the tracker as it had become their primary means of telling time. Activity tracking and coaching features were intrinsically enabling, leading some users to report that tracking had become part of their exercise routine.

The tracker kind of makes you conscious of what you’re doing.

Q: short-term user

### Barriers to Macrolevel Engagement

Some users had adopted a passive mindset in relation to the mHealth system, allowing steps to accrue throughout the day without monitoring their levels of activity. A few users only wore their trackers for structured exercise because they felt it was unfair to track incidental physical activity in the context of the NSC. Others experienced psychological pressure when using the mHealth system. Some users expressed disinterest in the rewards, and even a few sustained users felt they did not trigger any change in behavior. Other users openly questioned the morals of HPFIs.

[Concerning] rewards, I think it really depends on the person. Do they run because they want some reward or is it because of a more personal target? … I would say I run because I like to run, not for anything else.

J: sustained user

### Enablers of Macrolevel Engagement

Some sustained users internalized the NSC’s daily step target by developing interim targets that they could monitor throughout the day. Some described an autonomous process of setting personal step count or intensity targets that went beyond NSC thresholds. Others would even adopt a competitive mindset, either to outdo a previous level of activity or to outperform their peers. Most users reflected on HPFIs in transactional terms. This meant that the motivation to exercise and track their levels of activity diminished once the opportunity to earn vouchers had ceased. One participant, however, felt the incentives had outlived their purpose once he had become habituated to goal setting.

Right now, it’s kind of ingrained in me. Right now, I’m not even thinking about the vouchers. Right now, it’s just keeping fit. For me that’s the greatest reward.

C: sustained user

## Discussion

### Main Findings

Triangulation of methods uncovered that quantitative systems data alone painted an incomplete picture of active use among young men with obesity enrolled in the NSC. Our qualitative findings on barriers to microlevel and macrolevel engagement also demonstrated why a user might have failed to benefit from the NSC. Insights into enablers provided a mechanistic understanding of how the NSC initiated and inculcated new physical activity habits for a subset of users. Through further integration of findings, we shall now discuss the context of active use and engagement before examining how the NSC might have changed in physical activity behavior.

### Context of Active Use

Given the variety of wearable tracking systems and mHealth apps available outside the NSC, it was not implausible that users would consider switching systems [61,62]. It is possible that upgrades and patches to operating systems might have disrupted back-end data linkages. Device switching and other forms of offline use would therefore have contributed an apparent decline in quantitative active use even though the desired behaviors were still being produced [63]. Given this context, it follows that the subsequent interpretation of engagement could still be considered internally consistent despite what a strict interpretation of objective active use might have suggested [64].

### Context of Engagement

#### Self-monitoring and Goal Setting

Our findings have reinforced the notion that tracking devices on their own provided a feasible and acceptable means of self-monitoring and physical activity promotion [65]. While the NSC had prompted users to initiate, intensify, or extend physical activity [66], we suspect that generic performance targets might have also created a false ceiling in some sustained users [67]. Furthermore, it is plausible that inappropriate norms may have created negative feedback [68,69] or insecurity [70] among short-term users. Personalized goal setting, which has been a mainstay of physical activity promotion especially for weight loss maintenance, was exhibited by only a small number of users [71]. In this context, a shift in the goal setting strategy toward relative or personalized goals might enhance engagement.

#### Health Promoting Financial Incentives

At the macro level, health points had provided a virtual positive feedback loop that emulated customer loyalty program where membership, participation, and continued accrual of currency create their own intangible reward [72,73]. Even though only a minority of health points had been redeemed, interview data suggested that the system of HPFIs had communicated and reinforced a small but tangible external benefit of being physically active [74]. It was plausible that a less transactional or more attractive HPFI strategy (eg, a lottery [75] or endowment [76]) might have elicited higher enrollment, active use, and engagement.

### Lasting Behavior Change

Self-monitoring behaviors are thought to diminish at the end of an intervention [77] while goal setting strategies are considered more durable [78]. Once HPFIs had been discontinued, some sustained users may have stopped tracking because of a loss of so-called habitual exercise instigation [79]. This means that bidirectional tracking and physical activity behaviors were still contingent on HPFIs. Alternatively, facing the prospect of a definite end point, some short-term and sustained users might have preemptively dissociated from the NSC by going offline or switching devices. These actions demonstrated an intent to extend self-monitoring and goal setting behaviors and are consistent with habit formation [80,81]. An apparent decline in active use should therefore be anticipated in population health settings.

### Study Implications

Our study focused on a segment of the population that was not only at greater risk of the effects of inactivity, but also stood to reap real benefits from behavior change interventions such as the NSC [82]. These formative research findings have already aided our continuous efforts to promote physical activity in this group. We have also identified that pervasive offline use and device switching merit further investigation. Without due consideration for these phenomena, past studies that relied on quantitative mHealth data alone to categorize users [42] or to define nonuse attrition [43] might have inadvertently introduced misclassification biases that would have reduced the overall effect size or underestimated the public health impact of the interventions in question.

### Strengths and Limitations

Objective tracker data were triangulated with subjective interview data to improve the accuracy and validity of our findings. By focusing on the barriers and enablers to engagement, we were able to examine and discuss the varied context of lived experiences engaging in the NSC.

Our study was affected by several limitations arising from the choice of study population and protocol. The participants were recruited in the context of national service, thus limiting the generalizability of our findings. By conducting semistructured interviews near the workplace, we may have inadvertently introduced information biases that would have favored public initiatives. Moreover, social desirability biases may have prevented enrollees with low mHealth use from participating in the study.

### Conclusion

Our study described how young men with obesity experienced an mHealth initiative promoting physical activity. We recognized that a decline in active use had occurred in the context of tracker abandonment, offline use, widespread device switching, and occasional dissatisfaction with the NSC. Sustained users experienced engagement predominantly in the context of goal setting whereby HPFIs communicated the tangible benefit of a healthy lifestyle. As the NSC is implemented in this population, more emphasis should be placed on technical support and personalized activity targets to promote lasting behavior change.

## Appendix

Multimedia Appendix 1 Phases, procedures, and products of data collection and analysis.

Multimedia Appendix 2 Topic guide and associated codes for the conduct and analysis of semistructured interviews.

Multimedia Appendix 3 Qualitative and quantitative data outlining use of the national step challenge among n=29 study participants.

## Abbreviations

BMI: body-mass index
BREQ-3: Behavioral Regulation in Exercise Questionnaire 3
HPB: Health Promotion Board
HPFI: Health Promoting Financial Incentive
IPAQ-SF: International Physical Activity Questionnaire Short Form
mHealth: mobile health
NSC: national steps challenge
