# Supplementary figures and images for: Active Use and Engagement in an mHealth Initiative Among Young Men With Obesity: Mixed Methods Study
Source: JMIR Form Res. 2022 Jan 25;6(1):e33798. doi: 10.2196/33798 (PMC8826145; doi:10.2196/33798)

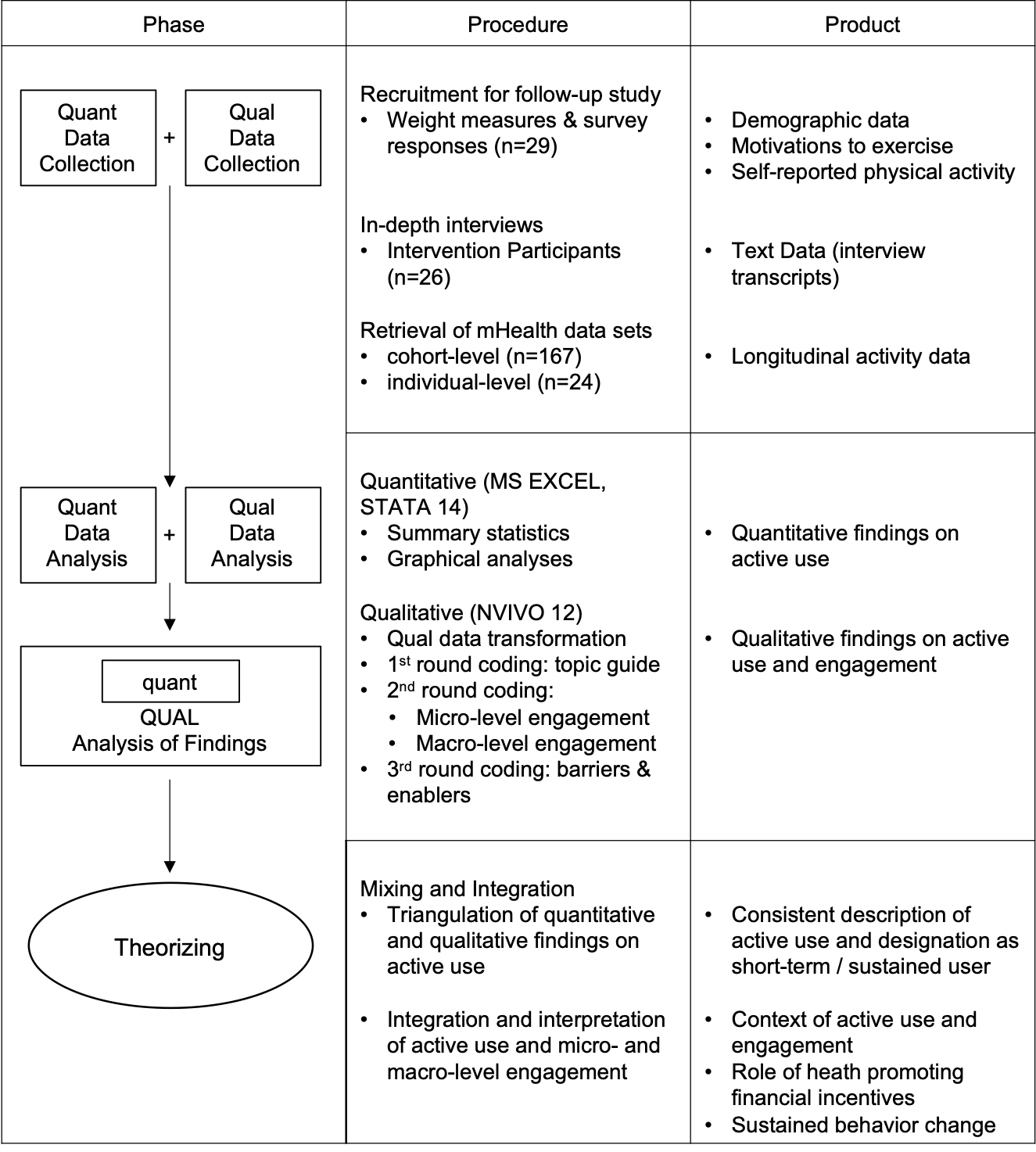

Supplement: Multimedia Appendix 1 [file formative_v6i1e33798_app1.png]
