# Supplementary material for: Active Use and Engagement in an mHealth Initiative Among Young Men With Obesity: Mixed Methods Study
Source: JMIR Form Res. 2022 Jan 25;6(1):e33798. doi: 10.2196/33798 (PMC8826145; doi:10.2196/33798)
Supplement: Multimedia Appendix 2 [file formative_v6i1e33798_app2.docx]

Multimedia Appendix 2. Topic guide and associated codes for the conduct and analysis of semi-structured interviews

|  | Material | Codes and sub-codes |
| --- | --- | --- |
| Introduction | - Familiarization – introduction of researchers and participant.  - Introducing the IDI format.  - Ground rules  - Stimulate discussion | NIL |
| Weight Management | - Do you track your own weight? | NIL |
| Physical Activity | - Where do you exercise?  - Do you exercise on your own? | **Reasons and motivations to exercise** |
| Diet / Nutrition | - How do your meals differ from the residential programme?  - Are healthy foods a part of your daily diet? | NIL |
| mHealth Use | - How did you find the National Steps Challenge (NSC) tracker? - Are you still tracking?  - How did you use the smart-phone application?  - What did you think about the rewards?  - What did you like about the NSC?  - Was there anything you disliked?  - Did the NSC change the way you behave? | **Barriers and Enablers to**  **A. Micro-level Engagement**  1. Trackers  2. Synchronizing data  3. Coaching prompts  **B. Macro-level Engagement**  1. Attitudes  - positive  - negative  2. Goal setting - self-monitoring - self-initiated exercise - attaining target  3. Rewards  - attractiveness of rewards - actual redemption |
| Conclusion | Remarks by facilitators. | NIL |
