# Supplementary material for: Active Use and Engagement in an mHealth Initiative Among Young Men With Obesity: Mixed Methods Study
Source: JMIR Form Res. 2022 Jan 25;6(1):e33798. doi: 10.2196/33798 (PMC8826145; doi:10.2196/33798)
Supplement: Multimedia Appendix 3 [file formative_v6i1e33798_app3.docx]

Multimedia Appendix 3. Qualitative and quantitative data outlining utilisation of the NSC among n=29 study participants

|  | Interview data (transformed qualitative) | Systems data (quantitative) | | | User Designation |
| --- | --- | --- | --- | --- | --- |
| Participant | Self-reported adherence | Number of valid^ active days during period of review (%) | Median  daily step-count (IQR) | Points awarded* for step-count activity |  |
| A | ≥ 4 months | 136 (89.5) | 12,573 (10,490; 15,445) | 4750 | sustained |
| B | ≥ 4 months | 119 (83.8) | 10,617 (6415; 14,384) | 3305 |  |
| C | ≥ 4 months | 119 (94.4) | 14,410 (9,989; 17,601) | 4065 |  |
| D | ≥ 4 months | 82 (81.2) | 12,842 (10,614; 16,027) | 2840 |  |
| E | ≥ 4 months | 74 (83.1) | 9,490 (6,068; 14,481) | 1860 |  |
| F | ≥ 4 months | 71 (86.6) | 6,796 (4,827; 9,711) | 1190 |  |
| G | ≥ 4 months | 56 (41.5) | 7,601 (6,162; 13,018) | 1190 |  |
| H | ≥ 4 months | 54 (88.5) | 12,751 (10,507; 15,833) | 1835 |  |
| I | ≥ 4 months | 41 (66.1) | 6,715 (8,469; 10,983) | 945 |  |
| J | ≥ 4 months | - | - | - |  |
| K | 1 to 3 months | 76 (70.4) | 14,830 (10,563; 17,305) | 2620 |  |
| L | 1 to 3 months | 54 (72.0) | 7,735 (5,712; 10801) | 1170 |  |
| M | 1 to 3 months | 31 (71.6) | 10,115 (4,666; 15,622) | 800 | short-term |
| N | 1 to 3 months | 10 (47.6) | 5,777 (4,968; 69,48) | 85 |  |
| O | 1 to 3 months | 0 | - | 0 |  |
| P | 1 to 3 months | - | - | - |  |
| Q | 1 to 3 months | 0 | - | 0 |  |
| R | 1 to 3 months | 0 | - | 0 |  |
| S | up to 1 month | 1 (7.7) | 4,730 | 0 |  |
| T | up to 1 month | 0 | - | 0 |  |
| U | up to 1 month | - | - | - |  |
| V | up to 1 month | 0 | - | 0 |  |
| W | up to 1 month | 0 | - | 0 |  |
| X | up to 1 month | - | - | - |  |
| Y | up to 1 month | 0 | - | 0 |  |
| Z | up to 1 month | - | - | - |  |
| AA | - | 137 (99.3) | 10,904 (8,181; 14,129) | 4180 | sustained |
| AB | - | 9 (90.0) | 13,476 (9,251; 15,385) | 315 | short-term |
| AC | - | 0 | - | 0 |  |
| Total | | 1,070 (78.8) |  | 31,150 | - |

^ Valid days defined as day when step-count ≥1,500

* Step-count cut-offs for points assigned by NSC:
5,000 – 7,499 steps/day (10 points); 7,500 – 9,999 steps/day (25 points); ≥10,000 steps/day (40 points)
